# The Longitudinal Relationships Between Sense of Place and Pro-Environmental Behavioral Tendencies of Adolescents: Evidence from Chinese Secondary School Students

**DOI:** 10.3390/bs16101732

**Published:** 2026-09-23

**Authors:** Xin Ai, Chenjing Lu, Yushan Duan

**Affiliations:** 1 School of Geographic Sciences, East China Normal University, Shanghai 200241, China; aixin@geo.ecnu.edu.cn; 2 National Research Center for Geographical Educational Materials, Shanghai 200241, China; 3 School of Geography and Environment, Jiangxi Normal University, Nanchang 330022, China

**Keywords:** sense of place, pro-environmental behavioral tendencies, longitudinal study, cross-lagged panel design, adolescence, secondary school students, China

## Abstract

To examine the longitudinal and reciprocal associations between adolescents’ sense of place and pro-environmental behavioral tendencies, this study employs a cross-lagged panel design to investigate the bidirectional relationship between these constructs. A total of 380 Chinese lower secondary school students completed the Environmental School Map task at two time points. Responses to the performance task were calibrated using a Many-Faceted Rasch Model (FACETS), and reciprocal longitudinal associations were examined using a cross-lagged panel model in Mplus. The results indicated that sense of place at Time 1 positively predicted pro-environmental behavioral tendencies at Time 2 (β = 0.177), while pro-environmental behavioral tendencies at Time 1 likewise positively predicted subsequent sense of place (β = 0.235). Further analysis using the cross-lagged panel model showed that both reciprocal associations remained significant after accounting for the temporal stability of each construct. Taken together, the findings provide evidence of a reciprocal longitudinal association between sense of place and pro-environmental behavioral tendencies during early adolescence and may have implications for school-based environmental education and place-based curriculum design.

## 1. Introduction

Global environmental challenges have intensified the need to understand the psychological foundations of pro-environmental behavior. In response, environmental education has become increasingly institutionalized in national curricula worldwide as a key strategy for promoting sustainable development (United Nations, 2015). This development reflects a growing international consensus that the cultivation of environmentally responsible citizens should begin during compulsory education. However, little is known about the psychological processes through which educational experiences translate into enduring pro-environmental behavior during adolescence. Against this background, examining the psychological processes underlying adolescents’ pro-environmental behavior is both necessary and important.

Pro-environmental behavior refers to intentional actions that minimize negative impacts on the natural environment and represents a key behavioral outcome of environmental education (Stern, 2000; Steg & Vlek, 2009; Maleknia & Zamani, 2025). Empirical research has demonstrated that environmental education can promote environmental engagement by enhancing knowledge, awareness, and concern (Zsóka et al., 2013; Vicente-Molina et al., 2018; Barth et al., 2021; Tian et al., 2025; Zhou et al., 2025). Especially, participation in nature-based learning and sustainability practices can further strengthen environmental engagement (Ting & Cheng, 2017; Collado et al., 2020). Nevertheless, research remains limited regarding how adolescents develop enduring pro-environmental behavior, highlighting an important area for further investigation.

Research on adolescents’ pro-environmental behavior has primarily focused on its social-psychological determinants and methodological assessment. Regarding determinants, family norms and parental modeling (Grønhøj & Thøgersen, 2012; Collado et al., 2019), as well as peer influences and school environments (Collado et al., 2019; Mackay & Schmitt, 2019; Ramkissoon, 2020; Bartolo et al., 2023), have been identified as important factors. Regarding measurement, researchers have developed both self-report and performance-based measures to assess pro-environmental behavioral tendencies (Blok et al., 2015; Lange et al., 2018; Lange & Dewitte, 2022; Otto et al., 2019; Chawla, 2020). However, these studies have largely approached the predictors of pro-environmental behavior in terms of internal and external factors, with comparatively less attention given to the psychological processes that connect adolescents’ everyday environmental experiences with sustained pro-environmental action (Colombo et al., 2023). Sense of place offers a theoretically grounded perspective for addressing this gap by conceptualizing how emotional, cognitive, and functional bonds with local environments may motivate environmental behavior over time (Tuan, 1979, 1990; Stedman, 2002; Collado et al., 2020).

Existing research has predominantly conceptualized sense of place as a psychological antecedent of pro-environmental behavioral tendencies (e.g., Vorkinn & Riese, 2001; G. Kyle et al., 2004; Stedman, 2003, 2008; Frimer et al., 2011; Milfont & Sibley, 2012; Krettenauer et al., 2024). Individuals who develop stronger emotional and identity-based bonds with places actively engage in environmentally responsible behaviors (Tam, 2013; Korpela et al., 2014; Restall & Conrad, 2015; Bartolo et al., 2023). However, emerging evidence suggests that ecological engagement may itself cultivate and reinforce sense of place through repeated behavioral practice and lived environmental experiences (e.g., Chow & Healey, 2008; Rollero & De Piccoli, 2010; Nussbaum, 2011; Ramkissoon et al., 2012; Whitburn et al., 2020). This possibility points to a potentially reciprocal relationship between sense of place and pro-environmental behavioral tendencies, yet empirical evidence remains limited. Most existing studies have treated this relationship as unidirectional, while the available evidence is predominantly based on cross-sectional designs, particularly among adolescents (Restall & Conrad, 2015; Krettenauer et al., 2024; Sun et al., 2024).

To strengthen the empirical evidence on the longitudinal associations between adolescents’ sense of place and pro-environmental behavioral tendencies, this study employs a two-wave cross-lagged panel design to examine their temporal and reciprocal relationships among Chinese lower secondary school students. Specifically, the study examines whether sense of place predicts subsequent pro-environmental behavioral tendencies, whether pro-environmental behavioral tendencies predict subsequent sense of place, or whether both processes operate simultaneously during early adolescence. Furthermore, the study may offer theoretical insights into environmental education and adolescent mental health development while advancing understanding of the psychological links between place and environmental behavior.

## 2. Literature Review

### 2.1. Sense of Place

Sense of place is a multidimensional psychological construct describing the affective, cognitive, and functional bonds individuals develop with meaningful environments. Although the concept has been widely applied across psychology, sociology, geography, environmental studies, and education, its conceptual origins are commonly traced to Tuan’s (1979, 1990) theory of topophilia, which emphasizes affective bonds formed through sensory experience and lived interactions with places (Stedman, 2002; Collado et al., 2020).

Rather than a unitary phenomenon, sense of place is conceptualized as comprising three interconnected dimensions: place attachment, place dependence, and place identity (Tuan, 1990; Jorgensen & Stedman, 2001; Raymond et al., 2010; Masterson et al., 2017; Kudryavtsev et al., 2012; G. T. Kyle et al., 2014; Ryfield et al., 2019; Yemini et al., 2025). Among existing frameworks, Jorgensen and Stedman’s (2001) tripartite model is particularly influential and has been operationalized through a validated 12-item scale assessing these dimensions. Growing evidence indicates that stronger emotional bonds and place identity are associated with greater environmental responsibility and more frequent engagement in pro-environmental action (e.g., Israel, 2012; Kudryavtsev et al., 2012; G. T. Kyle et al., 2014; Daryanto & Song, 2021; Yemini et al., 2025). Accordingly, sense of place has increasingly been conceptualized as an outcome of human–environment interactions and a key psychological foundation linking human–environment relationships to environmental engagement. This perspective provides a theoretical basis for examining its longitudinal associations with adolescents’ pro-environmental behavioral tendencies.

### 2.2. Pro-Environmental Behavioral Tendencies

Pro-environmental behavior refers to intentional actions aimed at protecting the environment, driven by environmental awareness and a sense of behavioral responsibility (Stern, 2000). As a central concept in environmental psychology, it helps understand how individual psychological processes contribute to sustainable development beyond technological and policy interventions (Gifford, 2014; Sawitri et al., 2015; Batool et al., 2024). Common models of pro-environmental behavior draw on three main theoretical frameworks: the theory of planned behavior (Ajzen, 1991; Lee et al., 2023; Gansser & Reich, 2023), the norm activation model (Schwartz, 1977), and the values–beliefs–norms (VBN) model (Stern et al., 1999; Han, 2015; Sharma & Gupta, 2020; Batool et al., 2024). According to these theories, pro-environmental behavior results from affective, motivational, and social-psychological processes that shape individuals’ willingness to act.

Several theoretical perspectives help explain the psychological foundations of pro-environmental behavioral tendencies. For instance, the biophilia hypothesis proposes that humans possess an inherent tendency to affiliate with nature, that contact with natural environments satisfies psychological needs and fosters positive affective responses, including place attachment, nature connectedness, and nature appreciation (Hinds & Sparks, 2008; Alcock et al., 2020; Zhou et al., 2025). Complementarily, interdependence theory (Davis et al., 2009) emphasizes that repeated interactions with natural and social environments shape social norms, environmental expectations, and behavioral intentions that further guide pro-environmental behavior (Thomas & Sharp, 2013; Farrow et al., 2017; Alcock et al., 2020; Q. Li & Wu, 2020; Teixeira et al., 2023; Zhou et al., 2025). Together, these perspectives suggest that pro-environmental behavioral tendencies develop through continuous interactions between individuals and their physical and social environments.

Adolescence represents a particularly important yet understudied period for understanding the psychological foundations of environmental behavior (Zylstra et al., 2014; Chawla, 2020; Bleidorn et al., 2022). During this stage, values, identities, and behavioral orientations remain highly malleable as adolescents gradually become less dependent on family influences and increasingly responsive to peers and broader social contexts (Steinberg, 2005; Somerville et al., 2010; Milfont & Sibley, 2012; Soenens et al., 2017; Ágoston et al., 2024). Simultaneously, urbanization has reduced opportunities for direct encounters with nature, potentially weakening adolescents’ environmental engagement and widening the gap between pro-environmental attitudes and behavior (e.g., Zylstra et al., 2014; Ojala, 2015; Olsson & Gericke, 2016; Soga & Gaston, 2016; Krettenauer et al., 2024; Ai et al., 2025). Moreover, empirical findings on developmental trajectories remain mixed: some studies report increasing environmental engagement with age (Otto & Kaiser, 2014), whereas others identify an “adolescent dip,” characterized by a temporary decline in the intensity of environmental engagement from early to middle adolescence (Kaplan & Kaplan, 2002; Olsson & Gericke, 2016; Krettenauer, 2017; Otto et al., 2019; Krettenauer et al., 2024). These inconsistencies underscore the need to better understand the psychological factors that support the development and maintenance of pro-environmental behavioral tendencies during adolescence.

### 2.3. Relationships Between Sense of Place and Pro-Environmental Behavioral Tendencies

A growing body of research has confirmed a positive association between sense of place and pro-environmental behavioral tendencies (Frimer et al., 2011; Milfont & Sibley, 2012; Krettenauer et al., 2024; J. Wang & Wang, 2026). Among the various psychological factors contributing to environmental engagement, sense of place has consistently been identified as a powerful predictor of pro-environmental behavioral tendencies, particularly during adolescence (e.g., Bonaiuto et al., 2002; Schultz et al., 2007; Devine-Wright, 2009; Kals & Müller, 2012; Korpela et al., 2014; Masterson et al., 2017; Mackay & Schmitt, 2019; Dang et al., 2022; Q. Wang et al., 2022). Individuals who develop stronger emotional bonds, place identities, and attachments to meaningful environments are more willing to protect local environments and engage in environmentally responsible actions (Sobel, 2004; Larson et al., 2018). More broadly, place-based connectedness has been associated with greater prosocial orientation, environmental concern, and pro-environmental engagement (e.g., Kudryavtsev et al., 2012; G. T. Kyle et al., 2014; Dreu et al., 2015; Rosa et al., 2018; Q. Wang et al., 2022; Chen et al., 2024). Thus, these findings suggest that sense of place functions as an important psychological antecedent of pro-environmental behavioral tendencies.

However, the association between sense of place and ecological engagement is neither unidirectional nor static. Individuals do not respond uniformly to places with which they are familiar or attached; rather, their environmental behaviors are shaped by the meanings they ascribe to those places (e.g., Vorkinn & Riese, 2001; G. Kyle et al., 2004; Korpela et al., 2014; Restall & Conrad, 2015; Bartolo et al., 2023). When valued places are perceived as threatened or degraded, individuals may be more likely to engage in protective or restorative behaviors (Krettenauer et al., 2020; Bartolo et al., 2023; Jiao et al., 2023; Sun et al., 2024). Conversely, repeated participation in pro-environmental activities can strengthen emotional attachment, place identity, and feelings of belonging by reinforcing individuals’ lived experiences of those places (Nussbaum, 2011; Ramkissoon et al., 2012; Whitburn et al., 2020; G. Li et al., 2024). Moreover, the durability and consistency of environmental engagement may play a critical role in shaping sense of place (Korpela et al., 2014; Erfani, 2022).

Although a growing body of research has demonstrated an association between sense of place and pro-environmental behavioral tendencies, several important gaps remain. First, most existing studies have relied on cross-sectional designs, which offer limited insight into the temporal ordering and potential directionality of the association between sense of place and pro-environmental behavioral tendencies. A cross-lagged design provides a stronger basis for examining the temporal ordering and prospective associations between these constructs. Second, previous research has predominantly conceptualized sense of place as an antecedent of pro-environmental behavioral tendencies, whereas the possibility that environmental engagement may, in turn, strengthen place-related psychological processes has received comparatively little empirical attention. Finally, although adolescence represents a critical developmental period for the formation of environmental values and behavioral orientations, longitudinal evidence from adolescent populations remains scarce. Therefore, this study employs a two-wave cross-lagged panel design to investigate the longitudinal reciprocal associations between sense of place and pro-environmental behavioral tendencies among adolescents.

## 3. Methodology

### 3.1. Participants and Procedures

This study was conducted in four public lower secondary schools in Jiangxi Province, China, with a total enrolment of 650 students. A two-wave longitudinal design was employed to collect data from the same cohort at two measurement occasions separated by six months. At both waves, participants completed the task during regular classroom sessions under identical administration procedures. As all participants were minors, parental or legal-guardian consent was obtained prior to participation, and participation was voluntary.

The same students were invited to participate on both measurement occasions. Of the 403 students initially recruited, 380 completed both the Time 1 (T1) and Time 2 (T2) assessments, and their records were successfully matched across the two waves, yielding 380 longitudinal cases and a response rate of 94.29%. The remaining 23 students did not provide usable matched data across both measurement occasions and were therefore excluded from the longitudinal analyses. Where individual item responses were missing, missing data were handled using Full Information Maximum Likelihood (FIML) estimation in Mplus. Of the 380 participants, 213 (56.1%) were boys and 167 (43.9%) were girls. There were 159 students in Grade 7 and 221 in Grade 8. The participants were recruited from Beijing Normal University Affiliated School of Xinyu (n = 69), Xinyu No. 9 Middle School (n = 87), Xinyu No. 4 Middle School (n = 135), and Xinyu No. 5 Middle School (n = 89).

To situate the research within the local educational context, a literature review and informal conversations with teachers and students were conducted before the formal survey. These preliminary activities informed the study design and helped ensure that the assessed constructs were relevant to the everyday experiences of lower secondary students in the region. Between T1 and T2, students continued to receive their regular geography and environmental education curriculum. No additional intervention or researcher-designed environmental programme was implemented, allowing the observed longitudinal associations to reflect naturally occurring developmental changes rather than experimental effects.

### 3.2. Data Collection and Instruments

This study’s cross-lagged panel design accounts for prior levels of each construct while enabling the simultaneous examination of cross-lagged associations and temporal stability (Kenny, 1975; Prati et al., 2016; Mund & Nestler, 2019; Krettenauer et al., 2024). As such, it provides a rigorous framework for distinguishing behavioral tendencies from psychological orientations across developmental and transactional processes (Otto et al., 2019; Xu et al., 2022).

Data were collected using a paper-based performance task during regular classroom sessions. Teachers distributed the materials to participating students after obtaining informed consent and explaining the purpose of the study. Students then completed the task independently in approximately 15 min.

The primary instrument was the “Environmental School Map,” an open-ended, performance-based task developed and administered independently by our team to elicit students’ sense of place and pro-environmental behavioral tendencies (Appendix A). Rather than relying on self-report questionnaires, the task prompted students to represent their everyday school environment through drawings, symbols, and written annotations, thereby providing richer evidence of their cognitive, affective, and behavioral orientations toward their familiar campus. By allowing students to express place-related meanings and behavioral tendencies within an authentic environmental context, the task also reduced the influence of socially desirable responding and provided richer evidence of implicit environmental orientations (Cox et al., 2022; Jackson et al., 2026).

A systematic analytical rating scale was used to evaluate students’ responses (annotated maps) based on two core constructs: Sense of Place and Pro-Environmental Behavioral Tendencies. Raters evaluated these two components using a five-point Likert scale (1 = extremely uncharacteristic to 5 = highly characteristic), rating the extent to which each was represented in the students’ work.

Students’ psychological ties to the educational environment served as evidence for assessing their **sense of place**. More specifically, **place dependence** was coded when students referred to a specific aspect of the school as functionally important, relevant, or necessary for their activities. **Place identity** was identified when narrative or symbolic elements reflected the cultural, social, or individual significance of the school as a unique site. **Place attachment** was inferred from expressions of emotional belonging, pride, affection, or a desire to protect or maintain a connection with the school.

In the present study, students’ pro-environmental behavioral tendencies were assessed through their performance on the Environmental School Map task. Map activities, narrative framing, and symbolic meanings were examined for evidence of **pro-environmental behavioral tendencies**. **Knowledge sharing** was identified when students actively communicated environmental facts or ecological concepts. Annotations that sought to persuade peers or others to adopt protective behaviors were coded as **persuasive actions**. Students who described or endorsed group- or community-based environmental practices were categorized as engaging in **civic action**. Finally, **behavioral intention** referred to students’ willingness to act to help preserve the environment, as inferred from their statements. More specifically, behavioral intention is conceptually distinct from actual behavior and represents a motivational antecedent to action, whereas pro-environmental behavioral tendencies constitute a broader construct encompassing different orientations toward pro-environmental action.

### 3.3. Rater Training and Scoring

Before formal scoring, the three evaluators underwent a rater training session (Appendix B). They received detailed explanations of the study objectives and the requirements for independent and impartial evaluation of the specified attributes. The scoring procedures and operational definitions for each attribute were also explained. Each construct comprised multiple dimensions: sense of place comprised three dimensions, whereas pro-environmental behavioral tendencies comprised four. Each dimension was rated on a five-point scale, with raw scores ranging from 1 to 5. Accordingly, the theoretical raw-score range was 3–15 for sense of place and 4–20 for pro-environmental behavioral tendencies. For each participant, the final raw score for sense of place was calculated by averaging the scores across the three dimensions, resulting in a theoretical range of 1–5. For pro-environmental behavioral tendencies, the final raw score was calculated by summing the scores across the four dimensions, resulting in a theoretical range of 4–20. Thus, the construct-level scores used in subsequent analyses were based on these summed raw scores.

To calibrate the scoring criteria, each rater practised scoring three typical maps that were excluded from the main sample. Any questions or disagreements concerning the criteria were discussed and resolved jointly to ensure consistent interpretation. Following training, each map was independently evaluated by all three certified evaluators. The scores assigned by the three evaluators were first averaged for each dimension within the two constructs. The resulting dimension scores were then aggregated according to the scoring rules described above. The FACETS-adjusted logits were used only for psychometric analysis of the rating process and were not used in the subsequent SPSS and Mplus analyses.

### 3.4. Psychometric Analysis

A Many-Faceted Rasch Model was estimated using FACETS Software (Version 3.8) to examine differences in rater severity and the functioning of the rating process, rather than to establish inter-rater agreement. The summed raw scores, rather than FACETS logits or transformed measures, were entered into SPSS and Mplus for subsequent correlation and cross-lagged analyses. The Many-Faceted Rasch Model simultaneously evaluates rater severity, task difficulty, and rating-scale functioning (Table 1). Specifically, separation reliability indicates how reliably the model distinguishes differences in rater severity rather than representing direct inter-rater agreement. The chi-square statistic tests whether differences in rater severity are statistically significant.

This integrated performance-based evaluation and measurement approach provided a psychometrically sound and ecologically valid means of capturing implicit attitudes toward place and behavioral intentions that may not be fully accessible through traditional self-report questionnaires.

### 3.5. Data Analysis

FACETS, SPSS 26.0, and Mplus 7.0 were used to analyze the data. The raw performance scores were initially examined using the Many-Faceted Rasch Model in FACETS (Version 3.8) to assess rater severity and calibrate the rating process. FACETS generated severity-adjusted logit measures based on the estimated rater parameters; however, these measures were not used as the final scores in subsequent statistical analyses. Instead, the summed raw scores derived from the original ratings were entered into SPSS and Mplus for the subsequent analyses.

First, descriptive statistics were calculated in SPSS to examine the central tendency and dispersion of all key variables. Pearson product-moment correlation coefficients were then calculated to assess the bivariate relationships between the research variables at T1 and T2.

Finally, a cross-lagged panel model was estimated using structural equation modeling in Mplus to examine the reciprocal longitudinal associations between sense of place and pro-environmental behavioral tendencies. The model specified autoregressive paths for each construct from T1 to T2, as well as reciprocal cross-lagged paths between the two constructs. Because participants were recruited from four schools and multiple classrooms, the TYPE = COMPLEX option in Mplus was used with school ID as the clustering variable to obtain cluster-robust standard errors. Moreover, sex, grade level, and age were included as covariates in a sensitivity analysis to assess the robustness of the cross-lagged associations.

## 4. Results

### 4.1. Descriptive and Correlation Analysis

Descriptive statistics for students’ sense of place and pro-environmental behavioral tendencies at both measurement occasions are presented in Table 2.

Partial correlation coefficients were also calculated to examine the relationships among the study variables (Table 2). After adjusting for sex, grade level, and age, sense of place was positively and significantly correlated with pro-environmental behavioral tendencies both concurrently at T1 (r = 0.615, *p* < 0.01) and at T2 (r = 0.758, *p* < 0.01). Both constructs also demonstrated significant temporal stability, as reflected in the within-construct correlations from T1 to T2 (sense of place: r = 0.357, *p* < 0.01; pro-environmental behavioral tendencies: r = 0.305, *p* < 0.01). In addition, the cross-time correlations between the two constructs were significant; for example, sense of place at T1 was positively correlated with pro-environmental behavioral tendencies at T2 (r = 0.357, *p* < 0.01). These preliminary findings establish the correlational basis for the subsequent analysis of the longitudinal cross-lagged associations between the two constructs.

### 4.2. Cross-Lagged Panel Analysis of Sense of Place and Pro-Environmental Behavioral Tendencies

Mplus 8.2 was used to estimate the cross-lagged panel model, with sense of place and pro-environmental behavioral tendencies as the primary constructs of interest. The model specified autoregressive paths from T1 to T2 for each construct, together with reciprocal cross-lagged paths between the two constructs, to examine their longitudinal associations (Figure 1).

The cross-lagged panel model was just-identified (df = 0; saturated model) and therefore yielded perfect fit indices (CFI = 1.00, TLI = 1.00, RMSEA = 0.00, SRMR = 0.00). As shown in Figure 1, sense of place at T1 positively predicted pro-environmental behavioral tendencies at T2 (β = 0.177, *p* < 0.005). Conversely, pro-environmental behavioral tendencies at T1 also positively predicted sense of place at T2 (β = 0.235, *p* < 0.001). However, because the model was saturated, the perfect fit indices are a mathematical consequence of the model specification and therefore do not provide substantive evidence regarding global model fit. Future intervention and longitudinal studies are needed to determine whether changes in these constructs translate into meaningful changes in students’ environmental engagement and related educational outcomes.

## 5. Discussion

### 5.1. Discussion of the Results

By constructing a two-wave cross-lagged panel model, this study extends predominantly cross-sectional research by examining the reciprocal longitudinal associations between sense of place and pro-environmental behavioral tendencies among Chinese lower secondary school students. The findings showed that sense of place at T1 positively predicted pro-environmental behavioral tendencies at T2, while pro-environmental behavioral tendencies at T1 likewise positively predicted sense of place at T2. These results suggest that sense of place and pro-environmental behavioral tendencies are reciprocally associated during early adolescence, providing longitudinal evidence that complements previous research highlighting the close relationship between place-based psychological processes and environmental engagement.

First, the cross-lagged panel model demonstrated that adolescents’ sense of place at T1 positively predicted their subsequent pro-environmental behavioral tendencies at T2. This finding is consistent with previous research identifying place-based psychological bonds as important antecedents of environmentally responsible behavior (e.g., Bonaiuto et al., 2002; Stedman, 2002; Devine-Wright, 2009; Kudryavtsev et al., 2012; Ramkissoon et al., 2012; G. T. Kyle et al., 2014; Dang et al., 2022; Zhang, 2025). From the perspective of the values–beliefs–norms model, pro-environmental behavioral tendencies develop through value-based beliefs and personal norms that motivate pro-environmental action (Stern et al., 1999; Q. Wang et al., 2022; Batool et al., 2024). Within this framework, sense of place may contribute to this prospective association by strengthening individuals’ psychological bonds with local environments and making environmental protection more personally relevant through ongoing interactions with those environments (Lewicka, 2011; Scannell & Gifford, 2017; Vada et al., 2019). Consequently, adolescents who develop stronger emotional connections to places they value may become more attentive to environmental quality and more willing to engage in behaviors that protect and preserve those environments (Larson et al., 2018; Dreu et al., 2015; Rosa et al., 2018; Daryanto & Song, 2021; Q. Wang et al., 2022; Chen et al., 2024).

Notably, the observed longitudinal association also supports the proposition that place-based psychological bonds possess a degree of temporal stability and persistence, shaping environmental orientations beyond immediate situational experiences (G. Kyle et al., 2004; Stedman, 2003, 2008; Raymond et al., 2010; Lewicka, 2011; Jiao et al., 2023; Lee et al., 2023). Once established, the core components of sense of place—place attachment, place identity, and place dependence—may function as relatively enduring psychological resources that remain associated with environmental concern and stewardship over time (Korpela et al., 2014; Scannell & Gifford, 2017; Q. Wang et al., 2022; Chen et al., 2024). The present findings therefore suggest that adolescents’ sense of place may represent a relatively stable psychological orientation that remains associated with subsequent pro-environmental behavioral tendencies. From an educational perspective, these findings imply that strengthening adolescents’ emotional and identity-based connections with local environments may support the development of more enduring pro-environmental engagement.

Second, the reciprocal pathway identified in the cross-lagged model, whereby adolescents’ pro-environmental behavioral tendencies at T1 positively predicted their sense of place at T2, has received considerably less empirical attention than the reverse pathway. The findings therefore extend previous research by suggesting that adolescents’ pro-environmental engagement may be not only an outcome associated with the development of sense of place but also a behavioral experience through which sense of place may subsequently develop (Chow & Healey, 2008; Rollero & De Piccoli, 2010). From the perspective of self-perception theory (Bem, 1972), repeated engagement in pro-environmental activities may gradually strengthen adolescents’ self-perceptions as environmentally responsible individuals, thereby reinforcing the emotional meanings they attach to the places in which these behaviors occur (Chawla, 2020; Goralnik et al., 2022). This interpretation is also consistent with research on nature connectedness and ecological identity, which suggests that sustained engagement with natural environments is associated with stronger ecological identity, place attachment, and psychological connectedness to place (Nussbaum, 2011; Ramkissoon et al., 2012; Whitburn et al., 2020; Erfani, 2022).

Therefore, through participation in environmental protection activities, adolescents may perceive improvements in the quality of their local environment while simultaneously developing stronger feelings of ownership, responsibility, and belonging toward those places (Mackay & Schmitt, 2019; Ramkissoon, 2020; Goralnik et al., 2022; Bartolo et al., 2023; Ai et al., 2025). Furthermore, environmental engagement has been associated with broader indicators of psychological well-being, including social connectedness, life satisfaction, and community belonging (Kasser, 2009; Ramkissoon et al., 2018; Suárez-Varela et al., 2016; Dang et al., 2022; Arbuthnott, 2023), which are conceptually related to the development of a stronger sense of place. Thus, the present finding suggests that providing adolescents with meaningful opportunities for environmental action may support the concurrent development of their sense of place and well-being.

Finally, the reciprocal longitudinal associations observed in the present study further underscore the dynamic nature of adolescents’ relationships with their changing environments. This interpretation is consistent with emerging research in environmental psychology suggesting that place meanings, environmental attitudes, and behavioral responses are continually reconstructed in response to changing social and environmental conditions (Bissell, 2021; Devine-Wright et al., 2020; McCunn, 2021; Bartolo et al., 2023). More broadly, major environmental and social disruptions can reshape individuals’ emotional connections with places and, in turn, influence their environmental attitudes and behaviors (Devine-Wright et al., 2020; Falanga, 2022; Ramkissoon, 2020). Conversely, repeated engagement in positive environmental actions may strengthen psychological attachment to place while fostering broader individual and community well-being (Ramkissoon, 2020; Dang et al., 2022; Arbuthnott, 2023). Although the present study did not directly examine these broader contextual processes, its reciprocal longitudinal findings are consistent with a dynamic perspective on human–environment relationships, suggesting that place-based psychological bonds and pro-environmental engagement may mutually reinforce one another throughout adolescence.

### 5.2. Implications

This study makes several theoretical contributions by examining reciprocal longitudinal associations between sense of place and pro-environmental behavioral tendencies among lower secondary school students using a two-wave cross-lagged panel design. The findings extend understanding of the dynamic relationship between place-based psychological bonds and environmental engagement during early adolescence. Rather than viewing sense of place solely as an antecedent of pro-environmental behavioral tendencies, the findings suggest that pro-environmental behavioral tendencies may also be associated with the subsequent development of adolescents’ place attachment, place identity, and place dependence. Thus, the findings support a more dynamic perspective on human–environment relationships, in which place-based psychological bonds and environmental engagement may reinforce one another over time.

From an educational perspective, the findings preliminarily suggest that place-based education and environmental engagement may be complementary rather than independent strategies. Schools may benefit from learning environments that strengthen students’ emotional connections with their schools and local communities while providing opportunities for meaningful environmental participation. Integrating these approaches may offer a promising direction for geography and environmental education by supporting the development of students’ sense of place and pro-environmental behavioral tendencies. However, future intervention studies are needed to determine whether such approaches can produce sustained changes in students’ psychological bonds with place and environmental engagement.

### 5.3. Limitations and Future Directions

Several limitations of this study should be acknowledged. First, participants were recruited from four lower secondary schools within a single province of China, which may limit the generalizability of the findings. Replication across different regions and cultural contexts are therefore needed to establish the broader applicability of the observed associations. Although cluster-robust standard errors were used to account for school-level clustering, the study did not explicitly model classroom- or school-level variation within a multilevel framework. In addition, the available results from the sensitivity analysis indicated that the directions and statistical significance of the primary cross-lagged associations remained unchanged after adjusting for gender, grade level, and age. However, these covariates do not account for other potentially relevant sources of confounding, such as family socioeconomic status and prior environmental experiences. Future research could employ multilevel modeling to examine more comprehensively how individual characteristics, such as personality traits (Liu et al., 2026), together with family- and school-level contextual factors, contribute to individual differences in the dynamic relationship between sense of place and pro-environmental behavioral tendencies.

Second, although the two-wave cross-lagged panel design enabled examination of the longitudinal associations and temporal ordering of the study variables, it cannot establish causal relationships or fully disentangle stable between-person differences from within-person changes over time. A random-intercept cross-lagged panel model (RI-CLPM) would provide a more rigorous framework for separating these components. Traditional CLPM may conflate these sources of variance (Hamaker et al., 2015); however, a complete RI-CLPM is generally not feasible with only two measurement occasions. Accordingly, the cross-lagged coefficients should be interpreted as prospective associations rather than evidence of individual-level causal developmental processes. Moreover, the two cross-lagged associations were not formally compared. Studies with more measurement occasions could therefore employ RI-CLPM and formal tests, such as Wald tests, to examine potential directional differences more rigorously. Although the Many-Faceted Rasch Model provided information on rater severity and rating-process functioning, conventional inter-rater agreement indices, such as ICC, were not available in the present study. Future research should incorporate complementary agreement measures alongside MFRM. Finally, the potential empirical overlap between sense of place and pro-environmental behavioral tendencies may partly reflect the use of the same open-ended Environmental School Map task to assess both constructs. Future research should examine their discriminant validity more directly and clarify the extent to which they represent distinct yet related dimensions of environmental orientation. Future studies should also report confidence intervals for the estimated coefficients and R^2^ values for the endogenous variables to provide more complete information on parameter uncertainty and model explanatory power.

Third, the six-month interval between measurement occasions may have been too short to capture meaningful developmental changes in adolescents’ sense of place and pro-environmental behavioral tendencies. The observed longitudinal associations should therefore be interpreted within this temporal window. Longer-term, multi-wave studies are needed to determine whether these reciprocal associations remain stable or change across development. In addition, the approximately 15-min completion time for the open-ended performance task may have constrained the depth of students’ responses and, consequently, the extent to which their cognitive, affective, and behavioral orientations were captured. Although the task was grounded in established theoretical dimensions of both constructs, its concurrent or convergent validity could not be directly assessed. Future studies could allow more completion time or develop more comprehensive versions of the task to obtain richer evidence.

Finally, future research should investigate the psychological and contextual processes underlying the reciprocal associations identified here. Environmental identity, emotional attachment, school climate, and social support may help clarify how sense of place and pro-environmental behavioral tendencies develop together during adolescence. As a newly developed performance-based measure, the Environmental School Map also assessed both constructs using the same task and evaluators, raising the possibility of shared-method variance and conceptual overlap. The relatively high concurrent association at T2 (r = 0.758) further underscores the need for multi-method validation using established self-report measures, behavioral observations, and other independent assessments, particularly because the Environmental School Map is a newly developed, open-ended task. Longitudinal intervention studies could additionally examine whether place-based educational programs strengthen adolescents’ psychological attachment and environmental engagement with their schools and communities over time.

## 6. Conclusions

This study provides longitudinal evidence of a reciprocal association between sense of place and pro-environmental behavioral tendencies among lower secondary school students. Findings from the two-wave cross-lagged panel model indicate that adolescents’ sense of place was positively associated with subsequent pro-environmental behavioral tendencies, while pro-environmental behavioral tendencies likewise predicted subsequent sense of place. These findings extend understanding of the dynamic relationship between place-based psychological bonds and environmental engagement during early adolescence, suggesting that sense of place is associated not only with subsequent environmental engagement but also with its own development alongside adolescents’ environmental engagement over time. This perspective contributes to environmental psychology by highlighting the reciprocal nature of human–environment relationships. From an educational perspective, the findings underscore the potential value of integrating place-based education with meaningful opportunities for environmental engagement. Early adolescence may represent a pivotal period for simultaneously strengthening emotional connections to local environments and fostering active environmental participation. Future longitudinal and intervention studies are needed to clarify the developmental processes underlying these reciprocal associations.

## Figures and Tables

**Figure 1:**
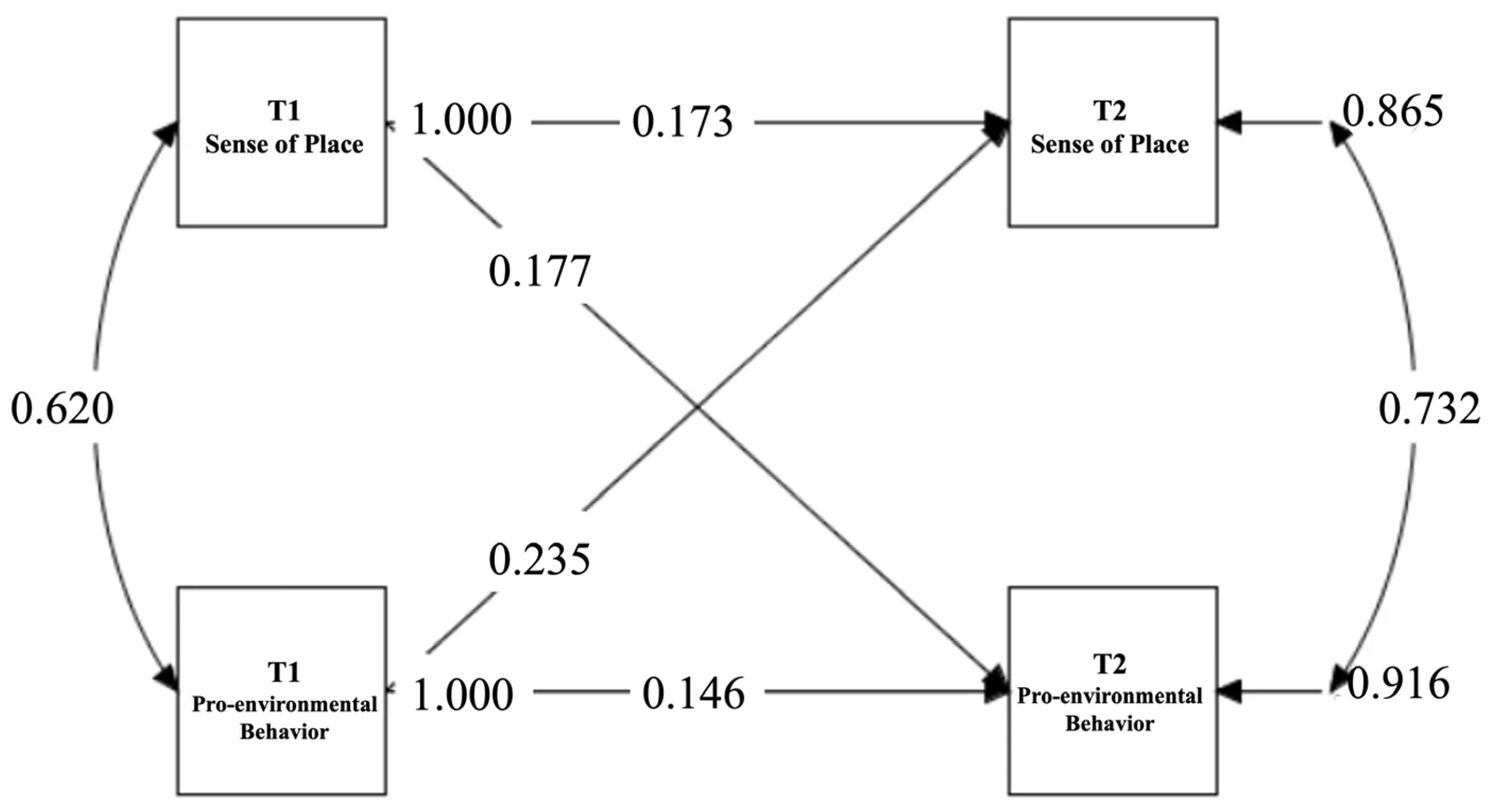
Cross-lag model of sense of place and pro-environment behavior.

**Table 1:** Summary statistics for the three-dimensional Rasch model analysis.

Facet	Reliability	Chi-Square (df)	RMSE	Separation	Strata	Measure	Model SE	Infit MnSq	Outfit MnSq	Obsvd Average	Fair (M) Average	Estim. Discrm
Evaluation indicator	0.73	23.6 (6) ***	0.18	1.66	2.55	0.00	0.18	1.02	0.99			
a. sharing knowledge						−0.46	0.17	1.19	1.69	1.29	1.35	0.74
b. persuasive actions						0.01	0.18	0.98	0.76	1.22	1.24	1.08
c. civic action						0.24	0.19	0.95	0.71	1.19	1.20	1.09
d. behavioral intention						0.43	0.20	0.92	0.57	1.17	1.17	1.16
e. place dependence						−0.46	0.17	1.19	1.69	1.29	1.35	0.74
f. place identity						0.01	0.18	0.98	0.76	1.22	1.24	1.08
g. place attachment						0.24	0.19	0.95	0.71	1.19	1.20	1.09
Rater	0.98	185.5 (2) ***	0.12	7.16	9.88	−2.50	0.12	1.03	0.99			
j1						−3.41	0.15	1.16	1.32	1.41	1.57	0.90
j2						−1.27	0.13	0.83	0.58	1.16	1.19	1.24
j3						−2.82	0.10	1.10	1.06	1.41	1.57	0.86

*** *p* < 0.001.

**Table 2:** Descriptive statistics and correlation analysis for sense of place and pro-environmental behavioral tendencies (n = 380).

Variables	M	SD	1	2	3
T1—Sense of Place	3.391	0.535			
T2—Sense of Place	3.993	0.856	0.357 **		
T1—Pro-Environmental Behavioral Tendencies	7.333	0.775	0.615 **	0.366 **	
T2—Pro-Environmental Behavioral Tendencies	9.000	1.007	0.357 **	0.758 **	0.305 **

** *p* < 0.01, T1 is the pre-test, T2 is the post-test. “1”, “2”, and “3” represent the variable numbers.

## Data Availability

The data supporting the findings of this study are available from the corresponding author upon reasonable request.

## Appendix A. Environmental School Map

Please design a campus environmental map within 15 min based on your observations and understanding of the school environment. You may use labels, text, drawings, symbols, tables, or other appropriate forms of presentation to represent features or information you consider important. Where appropriate, include relevant human and natural environmental features and indicate their relationships.

## Appendix B. Scoring Criteria

Each Environmental School Map was independently evaluated by three trained raters using a five-point scale for each indicator. Ratings reflected the amount and quality of observable evidence: 1 indicated no or minimal evidence, 2 limited evidence, 3 clear but moderate evidence, 4 strong evidence, and 5 particularly extensive and well-developed evidence. Raters based their judgments on the content, clarity, relevance, and development of students’ maps and annotations. Scores were assigned solely on observable evidence in the completed maps; raters did not infer information that was not explicitly or visually represented.

(**A**) **General Scoring Anchor**	
**Score**	**General Criterion**
1—Very low	The indicator is absent or minimally evidenced. Relevant content or action is unclear.
2—Low	Limited evidence of the indicator is present but incomplete, unclear, or poorly developed.
3—Moderate	Clear evidence of the indicator is present, but limited in detail, development, or integration.
4—High	Strong, clear evidence of the indicator is present, with good detail, development, and relevance.
5—Very high	Extensive, explicit, and well-developed evidence of the indicator demonstrates a particularly strong level of the construct.
(**B**) **Sense of Place**	
**Dimensions**	**Operational Criterion**
Place Dependence	The extent to which the student represents the school as fulfilling functions, activities, experiences, or needs that are meaningful or difficult to obtain elsewhere.
Place Identity	The extent to which the student represents the school as connected to personal or collective identity, self-definition, distinctive characteristics, or place-based meanings.
Place Attachment	The extent to which the student expresses emotional connection, affection, belonging, or attachment to the school and its environment.
(**C**) **Pro-Environmental Behavioral Tendencies**	
**Dimensions**	**Operational Criterion**
Sharing Knowledge	The extent to which the map communicates environmentally relevant knowledge, information, or understanding to others.
Persuasive Actions	The extent to which the map encourages or persuades others to engage in environmentally responsible behaviors.
Civic Action	The extent to which the map represents the student’s participation in, contribution to, or willingness to undertake environmental or collective action.
Behavioral Intention	The extent to which the map indicates the student’s willingness, intention, or tendency to engage in environmentally responsible behaviors.
